# Smallholder oil palm plantation sustainability assessment using multi-criteria analysis and unmanned aerial vehicles

**DOI:** 10.1007/s10661-023-11113-z

**Published:** 2023-04-17

**Authors:** Yong Bin Wong, Chris Gibbins, Badrul Azhar, Su Shen Phan, Paul Scholefield, Reza Azmi, Alex M. Lechner

**Affiliations:** 1 School of Environmental and Geographical Sciences, University of Nottingham Malaysia, 43500 Semenyih, Selangor Malaysia; 2 Faculty of Forestry and Environment, Universiti Putra Malaysia, 43400 Serdang, Selangor Malaysia; 3 Wild Asia, No 2, Jalan Raja Abdullah, 56000 Kuala Lumpur, Malaysia; 4 Centre for Ecology and Hydrology, Lancaster Environment Centre, Bailrigg, UK; 5 Monash University Indonesia, South Tangerang, 15345 Indonesia

**Keywords:** UAV, Oil palm smallholder, Sustainability assessment, Land cover and land use, Object-based image analysis, Multi-criteria analysis

## Abstract

Oil palm agriculture has caused extensive land cover and land use changes that have adversely affected tropical landscapes and ecosystems. However, monitoring and assessment of oil palm plantation areas to support sustainable management is costly and labour-intensive. This study used an unmanned aerial vehicles (UAV) to map smallholder farms and applied multi-criteria analysis to data generated from orthomosaics, to provide a set of sustainability indicators for the farms. Images were acquired from a UAV, with structure from motion (SfM) photogrammetry then used to produce orthomosaics and digital elevation models of the farm areas. Some of the inherent problems using high spatial resolution imagery for land cover classification were overcome by using texture analysis and geographic object-based image analysis (OBIA). Six spatially explicit environmental metrics were developed using multi-criteria analysis and used to generate sustainability indicator layers from the UAV data. The SfM and OBIA approach provided an accurate, high-resolution (~5 cm) image-based reconstruction of smallholder farm landscapes, with an overall classification accuracy of 89%. The multi-criteria analysis highlighted areas with lower sustainability values, which should be considered targets for adoption of sustainable management practices. The results of this work suggest that UAVs are a cost-effective tool for sustainability assessments of oil palm plantations, but there remains the need to plan surveys and image processing workflows carefully. Future work can build on our proposed approach, including the use of additional and/or alternative indicators developed through consultation with the oil palm industry stakeholders, to support certification schemes such as the Roundtable on Sustainable Palm Oil (RSPO).

## Introduction

Palm oil is the most widely traded and consumed vegetable oil, with derivatives that are common ingredients in many products. Rapid rates of fossil fuel depletion, along with disruptions to crude oil supply, are driving research programmes designed to develop palm oil as a biofuel source to support cleaner energy production (Johari et al., 2015; Tan et al., 2009). The growing demand for palm oil has led to the rapid expansion of plantations across tropical regions (Johari et al., 2015; Mekhilef et al., 2011; Xu et al., 2020), changing land use and land cover over large areas (Basiron, 2007; Shamshiri et al., 2019). In Malaysia, agriculture is one of the most economically important industries, with large-scale investment and widespread development of the plantation sector (Basiron, 2007). Malaysia is now one of the world’s largest palm oil producers and exporters (Azhar et al., 2017), with around 5 million ha of land in Malaysia currently under oil palm cultivation (Shamshiri et al., 2019).

The conversion of tropical forest and peatlands to oil palm plantations adversely affects the environment by reducing biodiversity and releasing greenhouse gases (Brandi et al., 2015; Carlson et al., 2018; Fitzherbert et al., 2008; Hawa et al., 2016; Shuhada et al., 2020; Vijay et al., 2016). Palm oil production also severely impacts air and water quality, giving rise to various public health issues (Carlson et al., 2018; Meijaard et al., 2020). However, the oil palm industry is also a profitable sector that generates positive socioeconomic outcomes and alleviates rural poverty by providing income opportunities to smallholder communities (Brandi et al., 2015; Rist et al., 2010). Consequently, there is a pressing need to promote sustainable production.

Interest in sustainable production has led to the development of certification standards for palm oil production, most notably the Roundtable on Sustainable Palm Oil (RSPO, 2018). Under the RSPO, growers adhere to a set of principles and criteria to ensure that palm oil is produced through environmentally responsible agronomic practices (RSPO, 2018). The certification system brings economic benefits to producers through access to international markets where there is demand for sustainable products (Brandi et al., 2015; Vijay et al., 2016). To improve sustainability of the industry as a whole, it is desirable for schemes such as the RSPO to be adopted by both large companies and smallholder farmers. Whilst large companies have resources and skills to improve practices and secure RSPO certification, smallholders frequently require external support (Saadun et al., 2018). Such support is being provided by a variety of agencies and initiatives, such as Wild Asia, an organisation whose goal is to help local communities implement sustainable agricultural practices. These schemes support farmers with their environmental commitments consistent with RSPO standards, including no cultivation in high conservation areas, mitigation of negative operational impacts on the environment, and implementation of best management practices for oil palm planting (Abdul Majid et al., 2021; RSPO, 2007). In addition, they support farmers more broadly by considering the human rights and the livelihoods of communities (Ruysschaert & Salles, 2017; RSPO, 2007). A key starting point for sustainable certification is to understand land use and land cover within plantations, and how agronomic practices affect these.

Satellite-based remote sensing technology is used in oil palm management to monitor land use cost-effectively (Chong et al., 2017). Remote sensing techniques can help to define boundaries between land cover types, thereby providing estimates of the oil palm planted area as well as spatial patterns in habitat type, integrity and connectivity, all of which are important ecologically. Furthermore, change detection techniques applied to remotely sensed time-series data help in the assessment of the potential environmental impacts of the expansion of oil palm plantations (Vijay et al., 2016). One of the drawbacks of satellite-based datasets is the presence of cloud cover, which masks crucial ground information and affects land cover mapping results (Chong et al., 2017; Shaharum et al., 2019; Vijay et al., 2016). Such cloud cover is a common hindrance in tropical countries such as Malaysia, limiting the value of satellite-based assessments.

Data collected from low altitude (i.e. below-cloud) using unmanned aerial vehicles (UAVs) are now used widely in precision agriculture (Liu et al., 2018, 2022; Shamshiri et al., 2019; Tsouros et al., 2019; Zhao et al., 2020) and provide a viable alternative to satellite data for oil palm mapping. UAVs are particularly suited to collection of high-resolution data for small areas, and so are ideal for the assessment of smallholder plantations (Chong et al., 2017; Kilwenge et al., 2021; Zhao et al., 2020). One of their particular advantages is that UAVs can rapidly acquire high spatial resolution images that can be used to discriminate different land cover classes effectively and accurately (Rokhmana, 2015; Yao et al., 2019; Zhao et al., 2020). They can also be deployed flexibly, so for example, the time-series data needed to assess temporal change can be more easily obtained than with satellite, especially where image capture at specific dates is required.

Considerable advances have been made recently to unmanned aerial platforms and various onboard sensors, leading to the proliferation and growing maturity of UAV-based remote sensing applications (Liu et al., 2022; Nex & Remondino, 2014; Pajares, 2015). UAVs can now be fitted with different and sometimes multiple sensors (Aslan et al., 2022; Feng et al., 2015; Kilwenge et al., 2021; Zhao et al., 2020), allowing simultaneous collection of complementary datasets useful in a wide range of applications (Kattenborn et al., 2014; Lechner et al., 2012; Yao et al., 2019). For instance, standard UAV-derived products, including orthophotos and three-dimensional geometric data, have been collected for urban infrastructure mapping and disaster management (Jia et al., 2022; Kerle et al., 2019), whilst UAV surveys that extract high spatial, spectral and temporal information have been applied in a range of environmental fields (Biggs et al., 2018; Feng et al., 2015; McKenna et al., 2017; Nowak et al., 2019). They have proven especially valuable in agriculture, for vegetation and crop mapping to support farm management and conservation (Chong et al., 2017; Liu et al., 2022; Pajares, 2015; Rokhmana, 2015). High spatial resolution digital surface models, which are common UAV-based products, can be integrated with optical sensor data to improve the accuracy of land cover mapping (Chong et al., 2017; Tsouros et al., 2019; Zhang et al., 2015). Ways of processing high-resolution UAV data sets have also developed and improved rapidly. For example, geographic, object-based image analysis (GEOBIA or OBIA) approaches are often applied by grouping similar pixels into image objects and then these image objects are classified (Blaschke et al., 2014; Liu et al., 2022; Tsouros et al., 2019; Yao et al., 2019).

Sustainable development is a complex, multidimensional issue, but ways of assessing it objectively are needed for policy development and evaluation (Boggia et al., 2018; Mohamadzadeh et al., 2020). Assessment remains challenging as sustainability indicators are very diverse by nature and often conflict (Milutinović et al., 2014; Mohamadzadeh et al., 2020). Multi-criteria analysis allows a variety of interrelated and conflicting indicators to be taken into account, and integrated to produce a single index value (Boggia et al., 2018; Mohamadzadeh et al., 2020). Multi-criteria analysis has been used extensively in strategic environmental planning and management, including assessment of sustainability (Boggia et al., 2018; Cinelli et al., 2014; Mohamadzadeh et al., 2020), so it is well-suited to oil palm landscapes.

The aim of this study was to develop and test a protocol for conducting sustainability assessments of smallholder oil palm production systems using UAV-derived data and multi-criteria analysis. The study had three objectives: (i) to assess the feasibility of using UAVs to map and classify land cover types on smallholder plantations, (ii) to apply multi-criteria analysis to the resulting land cover and land use data to create a map showing sustainability index values, and (iii) to use the sustainability index map to help prioritise management across the study area. A broader goal of the work was to assess the merits of combining the UAV mapping–based approach with multi-criteria analysis as part of evidence-based decision-making for agriculture.

## Materials and methods

### Study area

**Fig. 1:**
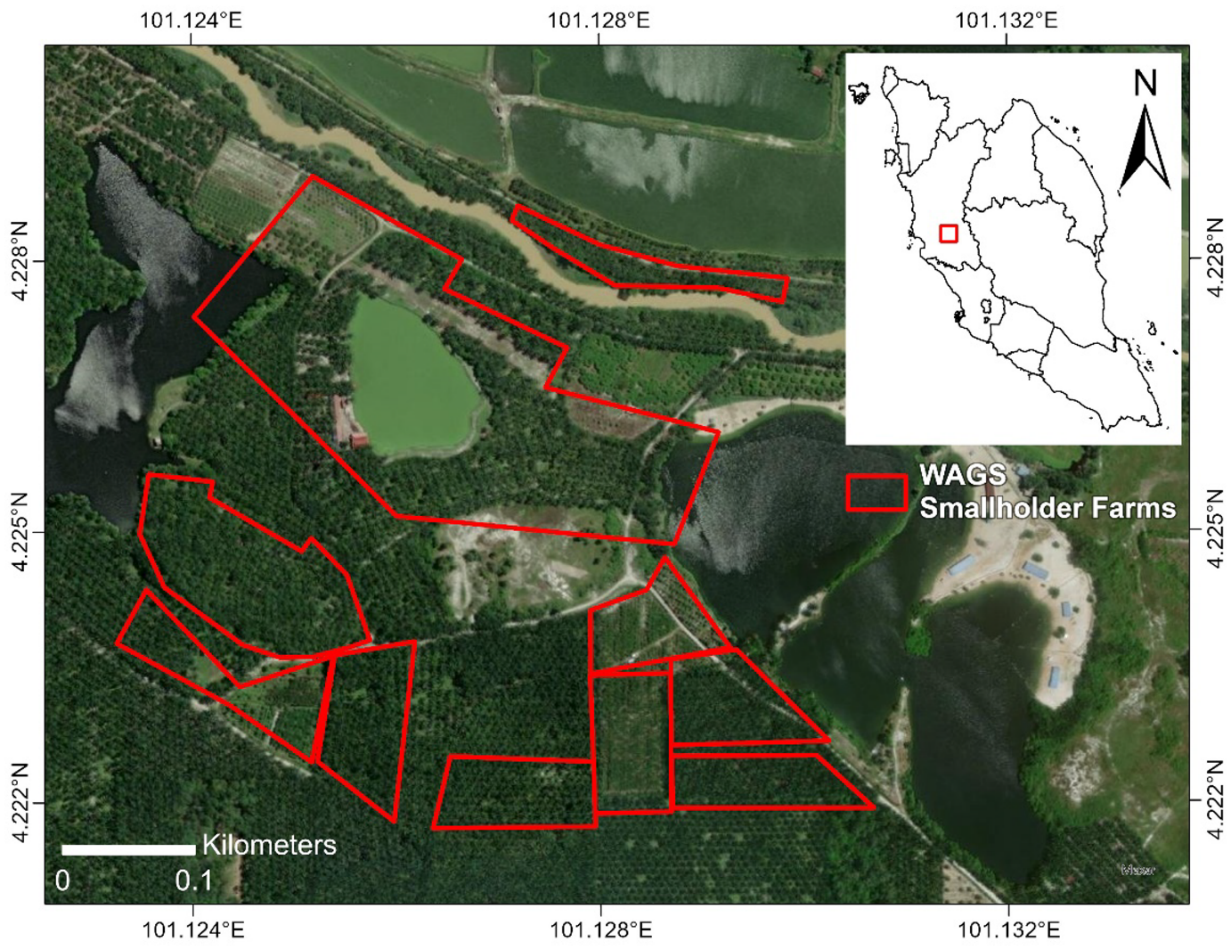
Location and details of the study area. Red areas are oil palm farms that are registered under the Wild Asia Group Scheme (WAGS)

The study area is situated in the Kampar district of Perak, Malaysia, and covers approximately 25 ha of oil palm farms registered under the Wild Asia Group Scheme (WAGS) (Fig. 1). The Wild Asia Group Scheme (http://oilpalm.wildasia.org/1030/wags/) or WAGS is a community development initiative working directly with smallholders to improve farming practices and achieve compliance with international certification standards, such as RSPO.

To capture the high landscape heterogeneity of oil palm smallholdings (Azhar et al., 2015; Ghazali et al., 2016; Sulai et al., 2015), an area with known smallholder farms adjacent to various biophysical features was selected for this study. During a site visit on 13 September 2019, the area was identified as heterogeneous, with a mixture of vegetation, unpaved roads, bare ground and water bodies. Vegetation consists predominantly of oil palms, but fruit trees, such as guava (*Psidium guajava*), banana (*Musa* spp*.*) and lime (*Citrus aurantifolia*), are also planted as commodity crops by the smallholders.

### Land cover classification

The first step in the land cover mapping was to develop a systematic classification scheme (Horning et al., 2010; Lechner et al., 2016). Amongst many conventional classification schemes, the United States Geological Survey (USGS) Land Cover/Land Use Classification System (Anderson et al., 1976) and the Food and Agriculture Organization (FAO) Land Cover Classification System (LCCS) (Gregorio & Jansen, 2000) are widely used, and both are hierarchically structured to offer flexibility by dividing broad-level classes into more detailed sub-classes (Congalton et al., 2014; Horning et al., 2010; Yang et al., 2017). This ensures land cover classes have appropriate spatial and thematic details suitable for specific user needs and requirements (Horning et al., 2010; Yang et al., 2017). Hence, a hierarchical approach was adopted in this study, with landscape features nested as a series of land cover and land use classes. This hierarchy also supported the remote sensing classification methods where coarser land cover classes were classified first and in the final steps the finer classes were identified.

**Fig. 2:**
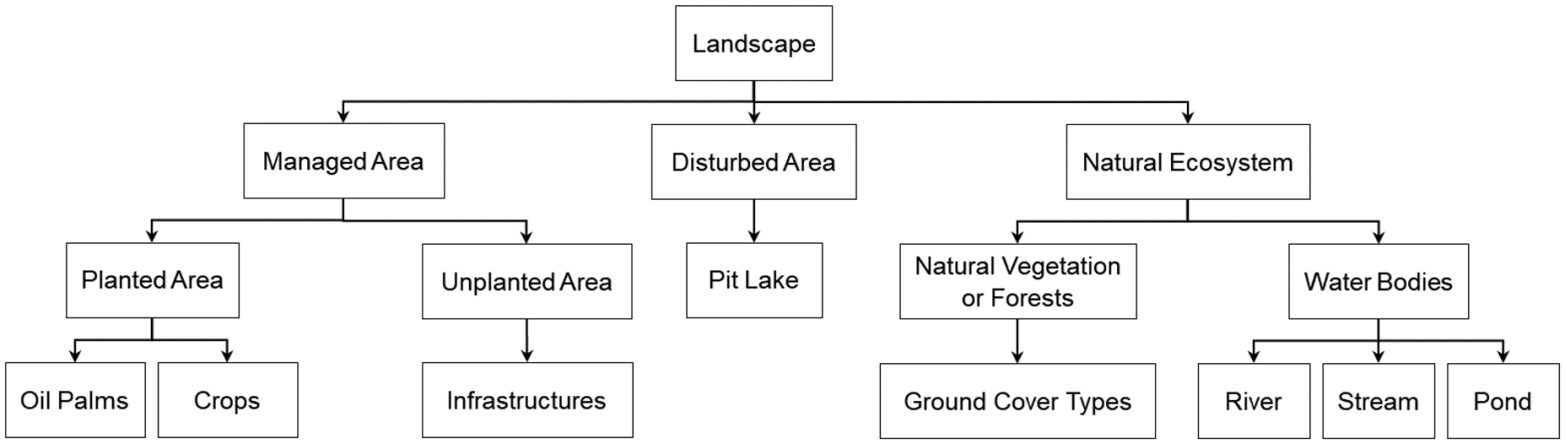
The hierarchical scheme used to classify land use and cover within the study area

The classification scheme was designed by first dividing the oil palm landscape into three broad land cover categories (Fig. 2): (i) natural ecosystems, (ii) areas that were managed by plantation owners and (iii) disturbed land from previous land use activities (e.g. sand mining, including lakes now formed in old sand mine pits). Fine-scaled sub-classes of the natural ecosystems comprised high carbon stock forests, high conservation value areas and water bodies, whereas areas managed by smallholders were divided into subcategories of plantation and non-plantation areas. Oil palm trees and crops were grouped under the plantation area, whilst buildings were considered non-plantation areas.

### Data collection

The surveys used a DJI Matrice 100 Quadcopter equipped with MAPIR Survey3W RGN (red, green, near-infrared) and Survey2 RGB (red, green, blue) sensors. Both sensors were attached beneath the UAV with nadir viewing direction. A GPS module was mounted to the UAV platform, and connected to the RGN sensor. Both sensors were connected to an intervalometer, which triggered their shutters simultaneously (2-s interval).

The flight mission was programmed to capture images 121 m above ground level, which is the maximum legal altitude for UAVs in Malaysia. Images were acquired with 80% frontal and side overlap. Five ground control points (GCPs) were distributed evenly across the survey area, each visible in the aerial images. The locations of GCPs were surveyed using a differential GPS with a nominal accuracy of 10 cm.

UAV data were collected on 13 September 2019. To minimise the impact of shadowed areas on image quality (Rahman et al., 2019; Stow et al., 2019), the flight was undertaken at noon. Flight paths were in a grid pattern, with a total flight distance of 14,059 m. The flight took approximately 40 min, with a total of 1269 images captured by each sensor.

### Pre-processing of UAV images

As the RGB sensor was not connected to the onboard GPS system during the aerial survey, images captured were geotagged manually. Images were pre-processed using the Pix4Dmapper v4.4.12 software, and the RGB and RGN imagery were used to produce a digital elevation model and orthomosaic of the surveyed area. During the initial processing stage, distinct features from the images were identified for computing keypoints, and tie points were generated for stitching images together. GCP locations were imported and marked using the keypoints to improve the quality of surface reconstruction. Once the GCPs were added, a quality report (see Figs. 12 and 13 in Appendix 1) was analysed to determine the parameters that need to be optimised.

**Fig. 3:**
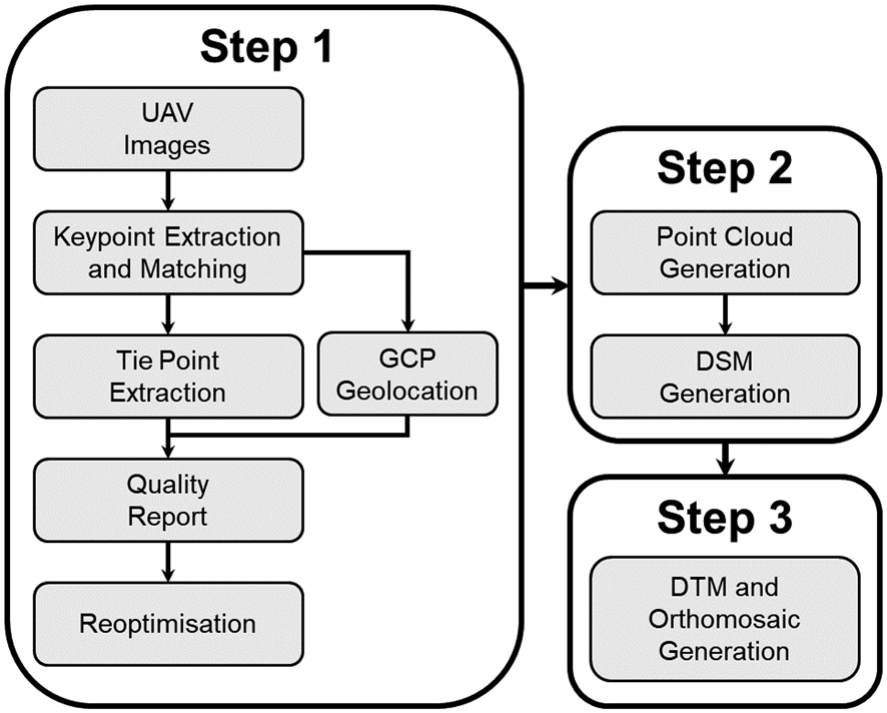
Schematic of image processing workflow to produce a digital surface model, digital terrain model and orthomosaic

Tie points were used to generate a 3D point cloud, which were subsequently used to derive a digital surface model (DSM). Structure from motion (SfM) photogrammetry with bundle adjustment was used to orthorectify the images, with well-defined geometric features from overlapping images used to create the point cloud needed for surface reconstruction (Hashemi-Beni et al., 2018; Wallace et al., 2016). Surface smoothing was applied to retain sharp features such as the edges of oil palm fronds and corners of buildings, whilst inverse distance weighting (IDW) interpolation was employed to generate the DSM. A digital terrain model (DTM) and orthomosaic were then generated from the point cloud and DSM. An overview of the pre-processing of UAV images is shown in Fig. 3.

### Texture analysis

Land cover classification using high-resolution imagery can be challenging. This is mainly because the high spatial resolution increases the spectral covariance and, therefore, reduces the spectral separability of land cover classes. The inherent complexity of landscapes also makes traditional spectral classification more difficult. Hence, conventional spectral analysis is often integrated with texture analysis. Texture analysis is a statistical measure of the structural properties of an image, and many studies have shown that its use can improve classification accuracy (Feng et al., 2015; Girolamo-neto et al., 2019; Laliberte & Rango, 2009). Texture analysis has been successfully applied to fine-resolution images in vegetation mapping, with different vegetation covers being discriminated based on textured surfaces. In this study, the near-infrared image data from the RGN orthomosaic were chosen for texture calculation as they had a high contrast that separated vegetated from non-vegetated areas. A texture image was created using Erdas Imagine v16.6 software, with the textural measure calculated within a moving window. Different textural measures with various window sizes were tested. Based on visual assessment, a texture image derived using mean Euclidean distance with 41 × 41 window size was adopted; this size yielded images with the detail and clarity needed to distinguish the vegetation classes present across the study area.

### Object-based image analysis (OBIA) classification

OBIA is often superior to pixel-based image analysis for classifying high-resolution imagery as object characteristics are no longer defined by a single pixel (Blaschke et al., 2014; Laliberte & Rango, 2009; Pande-Chhetri et al., 2017; Whiteside et al., 2011). Furthermore, OBIA methods overcome the so-called salt and pepper effect by categorising pixels into homogeneous objects based on their spatial, spectral and textural characteristics (Bao et al., 2014; Blaschke, 2010). The eCognition Developer software is widely used in remote sensing studies, with about 50% of OBIA studies using this software in 2010 (Blaschke, 2010). eCognition allows for the implementation of advanced segmentation algorithms (i.e. multiple scales, input data) within a rule-based and hierarchical classification workflow (Blaschke et al., 2006; Hossain & Chen, 2019; Ma et al., 2015) and is well suited to high spatial resolution UAV imagery and the hierarchical classification used in our study. The Fractal Net Evolution Approach with a multiresolution segmentation algorithm (Baatz & Schäpe, 2000), which is a bottom-up segmentation approach to merging pixels with similar characteristics, was selected for segmenting the RGN imagery. The segmentation of pixels was controlled by segmentation parameter scale, shape and compactness, with respective values of 40, 0.1 and 0.5, determined from visual examination.

**Fig. 4:**
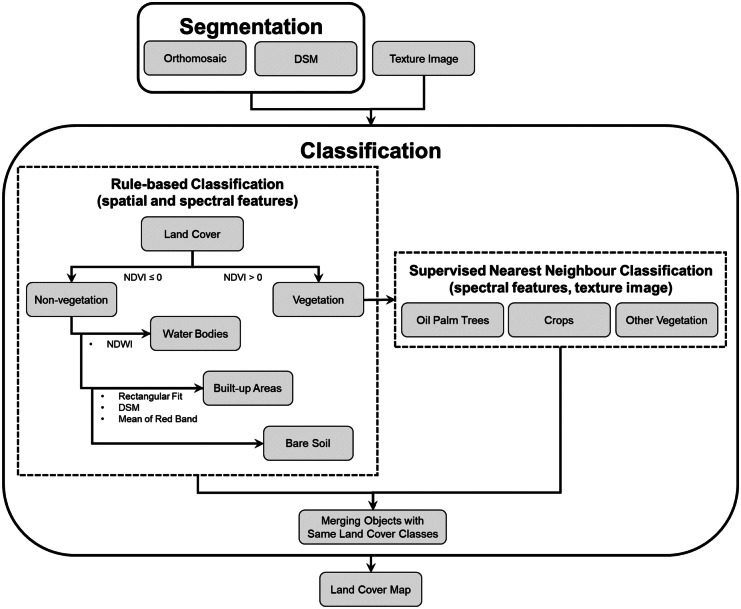
Method for object-based classification including a rule-based workflow using the eCognition software. The image classification was performed using the RGN orthomosaic and DSM with a texture image. DSM, digital surface model; NDVI, normalised difference vegetation index; NDWI, normalised difference water index

A rule-based classification was implemented in eCognition using threshold expressions for various spatial and spectral features used for the land cover classification analysis. Using the RGN orthomosaic, a set of spectral indices was computed to aid the classification, including the normalised difference vegetation index (NDVI) and the normalised difference water index (NDWI). NDVI is the normalised ratio between the near-infrared band and red band (Tucker, 1979), and is commonly used as an indicator for the greenness of vegetation and is useful for separating vegetation from non-vegetation (Chong et al., 2017; Srestasathiern & Rakwatin, 2014; Xue & Su, 2017), whilst NDWI is the normalised ratio of the green band and near-infrared band (McFeeters, 1996) and is used to delineate surface water features such as for monitoring water dynamics (Avdan et al., 2019; Ji et al., 2009). In this study, land cover was first divided into areas of vegetation and non-vegetation using the NDVI according to the hierarchy (Figs. 3 and 4). Then, the non-vegetated areas were subdivided into three further classes (water bodies, built-up areas and bare soil). Water bodies were identified using the NDWI, whilst built-up areas were classified based on shape, spatial and spectral features. Buildings are typically square-shaped objects with an elevation above ground (Jabari & Zhang, 2013; Tong et al., 2012); therefore, high rectangular fit and elevation were used in extracting building segments. Additionally, most buildings in the study area had a red-brown roof; thus, the red band values was incorporated into the process of extracting built-up areas. Once the water bodies and built-up areas were extracted, the remaining segments of the non-vegetation cover were then classified as bare soil.

As various types of vegetation have similar spectral characteristics, spectral features were incorporated in addition to texture image to classify oil palm trees, crops and other vegetation. A supervised classification was applied using the Nearest Neighbour classifier to identify different vegetation types, with about 30 training samples of each vegetation class selected for the classification analysis. Once the classification was completed, it was refined by merging objects with the same land cover class. Figure 4 shows the OBIA classification, applied using eCognition software.

### Accuracy assessment

An accuracy assessment was conducted using ArcGIS v10.6 to derive a confusion matrix based on a set of 45 randomly distributed points. The confusion (or error) matrix (Congalton, 1991; Foody, 2002) is a cross-tabulation of a classified map against validation samples from reference data. This matrix is commonly used to assess classification accuracy, and several accuracy measures were extracted from the error matrix table, including the overall accuracy, kappa coefficient, user accuracy and producer accuracy (Congalton, 1991; Foody, 2002; Olofsson et al., 2013). The validation points were identified through manual image interpretation of the original high-resolution RGN imagery.

### Sustainability assessment

The classified images were used to produce a sustainability map of the study area. The sustainability criteria used by the RSPO (2018) were modified to allow application to a site-scale spatial dataset (see Table 6 of Appendix 2). Many papers have described spatial patterns of biodiversity in oil palm landscapes and developed indicators that can be used to produce sustainability maps (Asmah et al., 2017; Ghazali et al., 2016; Syafiq et al., 2016; Tee et al., 2019).

Six environmental sustainability criteria were adopted (Table 1), with multi-criteria analysis integrated with the use of geographic information system (GIS) to generate individual criterion maps. These criteria represented five environmental indicators identified by previous work: (i) erosion protection on slopes, (ii) riparian vegetation, (iii) water clarity index, (iv) channel modification and (v) landscape connectivity and quality. Threshold values for sustainability for each of the criteria were identified with the aid of peer-reviewed papers and published reports (Baral et al., 2014; Fryirs & Brierley, 2012; Barclay et al., 2017; Standards Malaysia, 2006; Wild Asia, 2018). Threshold values were used to distinguish between low, medium and high sustainability, as detailed below.

**Table 1:** Indicators and criteria for assessing the environmental sustainability of smallholder land cover and land use

**Indicators**	**Criteria**	**Land cover categorised**	**Sustainability class (score)**		
			**Low (1)**	**Medium (2)**	**High (3)**
Erosion protection on slopes	Steepness of slopes with bare soil or oil palms (°)	Bare soil and oil palm trees	> 25	10–25	≤ 10
Riparian vegetation	Lack of natural vegetation within the riparian buffer of a river (m)	All land cover, except water bodies within 40 m of a river	≤ 20	20–30	30–40
	Lack of natural vegetation within the riparian buffer of a standing water body (m)	All land cover, except water bodies within 100 m of a standing water body	≤ 100	-	-
Water clarity index	Mean red band value of a water body	All water bodies	179.5–142.0	142.0–104.5	104.5–67.1
Channel modification	Channel sinuosity	River	1–1.05	1.06–1.30	> 1.31
Landscape connectivity and quality	Proximity to natural vegetation (m) multiplied with land cover coefficients	All land cover	> 50	50–25	≤ 25

The presence of risk of erosion due to bare earth on slopes was used as an indicator to represent the importance of minimising soil erosion (Standards Malaysia, 2006). Erosion risk was mapped by deriving a slope map from the digital elevation model, with the slope values for areas with bare soil and with oil palms categorised into three sustainability classes.

The second, third and fourth indicators are related to aquatic elements of the landscape. Riparian buffers serve numerous functions (Dwire & Lowrance, 2006; Wenger, 1999). Forested buffers are recognised as providing canopy cover and shading to the channel, which can be important for providing energy subsidies (e.g. in the form of leaf litter) (France et al., 1996) and to help moderate temperature extremes (Johnson & Jones, 2000) respectively. The presence of mature trees along riverbanks can also be important for stabilisation resulting from their extensive root systems (Beschta & Weatherred, 1984). Natural habitats in the riparian area can provide additional buffering effects, including filtering agricultural pollutants, trapping fine sediments and mitigating flood risk (Barclay et al., 2017). The clearance of natural areas and planting of oil palm or other crops all the way to the channel edge can therefore be considered a non-sustainable practice, so we analysed images to determine the presence of a buffer. Assessing buffer characteristics needed to provide protection, including precise width of the buffer needed, can be rather complex (Hilary et al., 2021; Lee et al., 2004) so we adopted a simple presence/absence approach. For the streams and rivers in the study area, we defined sustainable practice as being where a natural vegetation buffer greater than the channel width was left in place. To assess this, a buffer along the channel was first created in ArcGIS, which on each bank was the same width as the channel. Then, a continuous raster surface was created within a moving window with a neighbourhood of a radius equivalent to the channel width. For standing water bodies (ponds and lakes), we adopted the acceptable buffer width of 100 m suggested by Barclay et al. (2017) and mapped areas with and without this buffer.

Water clarity was used as an indicator of water quality. Water may become less clear (= more turbid) as a result of fine sediment inputs or the presence of abundant microalgae resulting from nutrient enrichment. Many studies have found a strong positive correlation between water turbidity and values in the red spectrum in RGB images (Joshi et al., 2017; Shen et al., 2021; Teo et al., 2021). Thus, red band values were extracted for all pixels across each water body and the mean value was used as an index of relative turbidity for each one. We assigned each water body to one of three water clarity classes, with classes based on the range of red band values observed across the whole of the study area. One of the water bodies was extremely clear, based on field surveys, and this had a mean red band value of 67.1. This was therefore taken as the baseline for the high sustainability class, with the other classes based on increasingly high red band values (see Fig. 14 and Table 7 in Appendix 3).

The fourth indicator represented physical modification of river and stream channels. A common practice in agricultural landscapes is the canelisation of river water courses, i.e. straightened and over-deepened, to aid water conveyance. As it can be calculated easily from aerial images, the sinuosity index was used as a measure of the degree of channel straightening. For each water course in the study area, sinuosity was computed by dividing the observed channel length between start and endpoints by the straight-line distance between respective points.

The fifth indicator used connectivity as a measure of the likely ecological integrity of areas. Natural areas have important ecological functions, such as providing habitats and food resources (Dislich et al., 2017), so connectivity to such areas is important. Connectivity was mapped by using the distance to natural vegetation multiplied with land cover coefficients (0, 0.5 or 1.0) that represented land cover quality, ecological and conservation values. A coefficient of 1 was assigned to natural vegetation and the river. On the other hand, bare soil and built-up areas that supported a few or no conservation values were assigned a coefficient of 0.

A final sustainability map was produced using a multi-criteria analysis approach with overlap analysis to provide an overall assessment of the oil palm landscape. For this, the weighted linear combination method was used for aggregating scores. This is one of the commonly used aggregation operators (Ghajari et al., 2017; Jeong & Ramírez-Gómez, 2017; Mehri et al., 2019). It requires the indicators to be weighted based on their relative importance, with all weighted criterion layers, and then summed to yield a composited layer (Ghosh & Lepcha, 2019; Mehri et al., 2019; Pérez et al., 2005). In this study, all indicators were considered to have equal importance in contributing to farmland sustainability, so they were weighted equally. Their respective indicator maps were then combined to generate a single composite sustainability map for the study area. This map showed the spatial distribution of sustainability values within the oil palm plantation areas, with high values representing more sustainable areas. Areas with low sustainability scores were taken to be priority areas for adopting more sustainable practices and/or improving existing conditions.

## Results

### DSM, DTM and orthomosaics

**Fig. 5:**
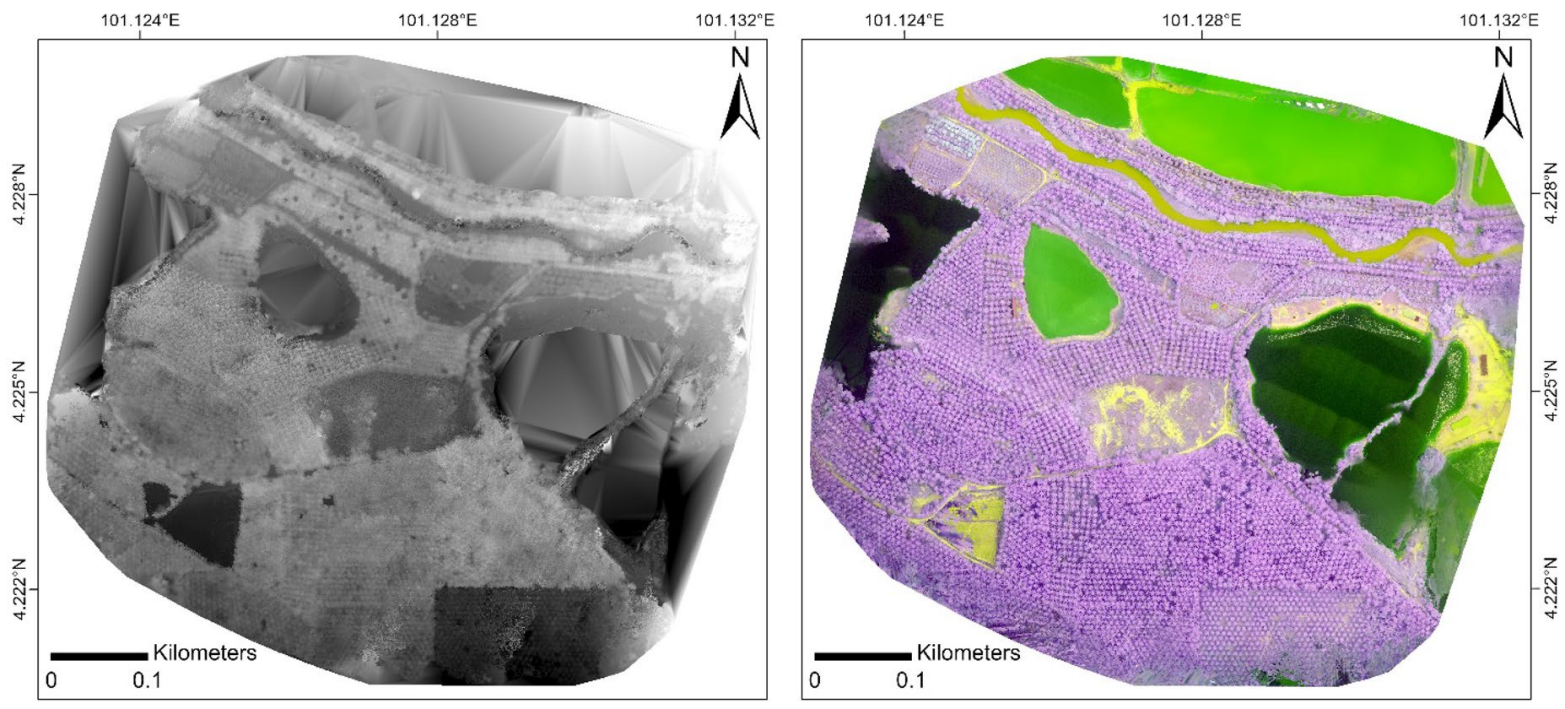
DSM (left) and orthomosaic (right) generated from RGN image dataset

**Fig. 6:**
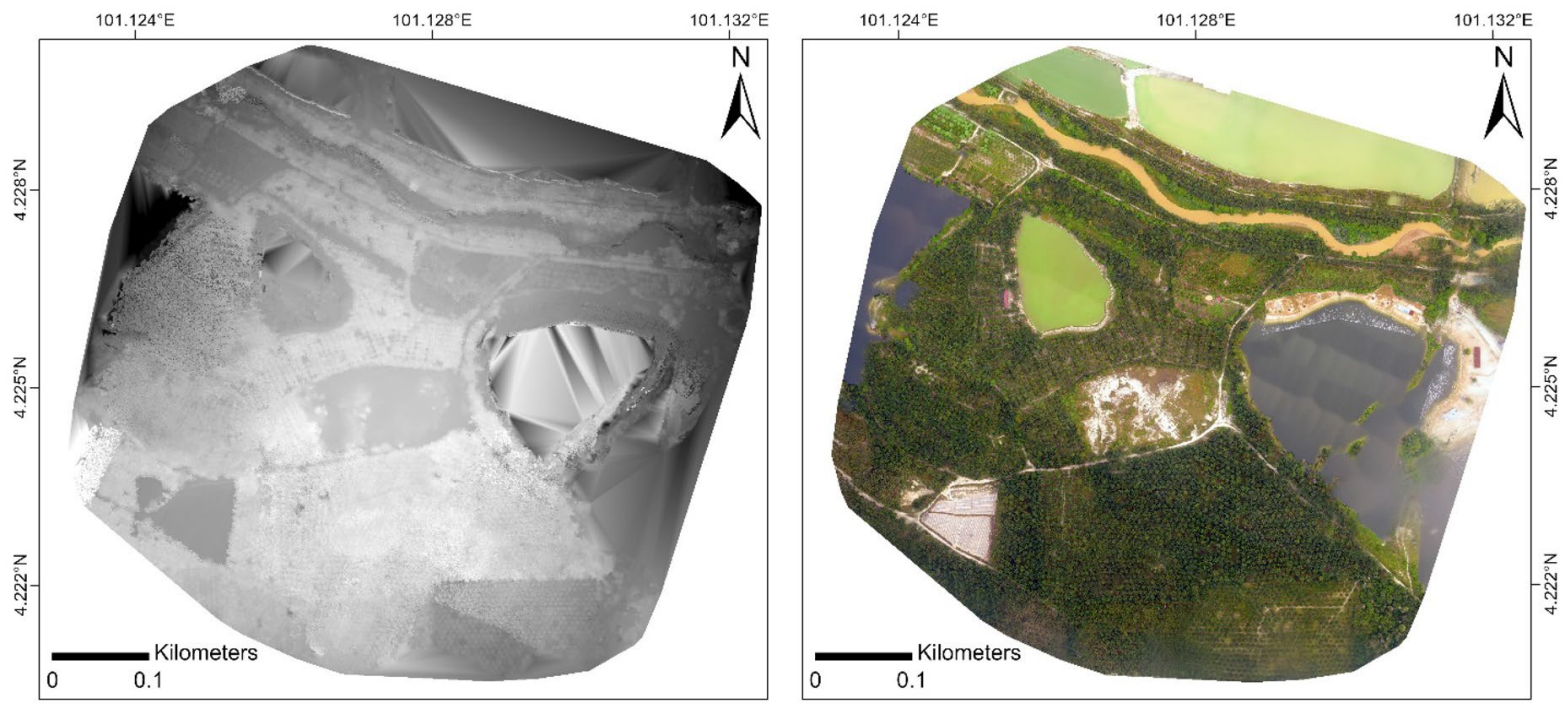
DSM (left) and orthomosaic (right) generated from RGB image dataset

Based on the quality report generated by Pix4D (see Appendix 1), the RGN image dataset had an average ground sampling distance (GSD) of 5.59 cm, whilst the RGB image dataset had a GSD of 4.56 cm. The root-mean-square error (RMSE) values of the RGN and RGB imagery were 0.026 m and 0.007 m, respectively (Table 2). The DTM of the RGN and RGB imagery had a corresponding resolution of 27.95 cm and 22.80 cm; the RGN orthomosaic and DSM (Fig. 5) had a spatial resolution of approximately 22 cm, whereas those generated from the RGB image dataset (Fig. 6) had a resolution of around 18 cm.

**Table 2:** Summary of results of pre-processing of UAV images

		**RGN**	**RGB**
Ground sampling distance (GSD) (cm)		5.59	4.56
Calibrated images (%)		98	95
Single/multiple blocks		2 blocks	4 blocks
Mean keypoints/image		4,598	5,637
Matching (matches/calibrated image)		1,500	992
Georeferencing		Yes, 5 GCPs	Yes, 5 GCPs
2D keypoints bundle block adjustment		1,438,374	939,838
3D point bundle block adjustment		614,785	428,846
Mean RMSE value (m)		0.026	0.007
**-**	*X*_RMSE_ (m)	0.034	0.009
**-**	*Y*_RMSE_ (m)	0.008	0.003
**-**	*Z*_RMSE_ (m)	0.041	0.008
Orthomosaic and DSM resolution (cm/pixel)		22.36	18.24
DTM resolution (cm/pixel)		27.95	22.80

### Land cover map and classification accuracy

**Fig. 7:**
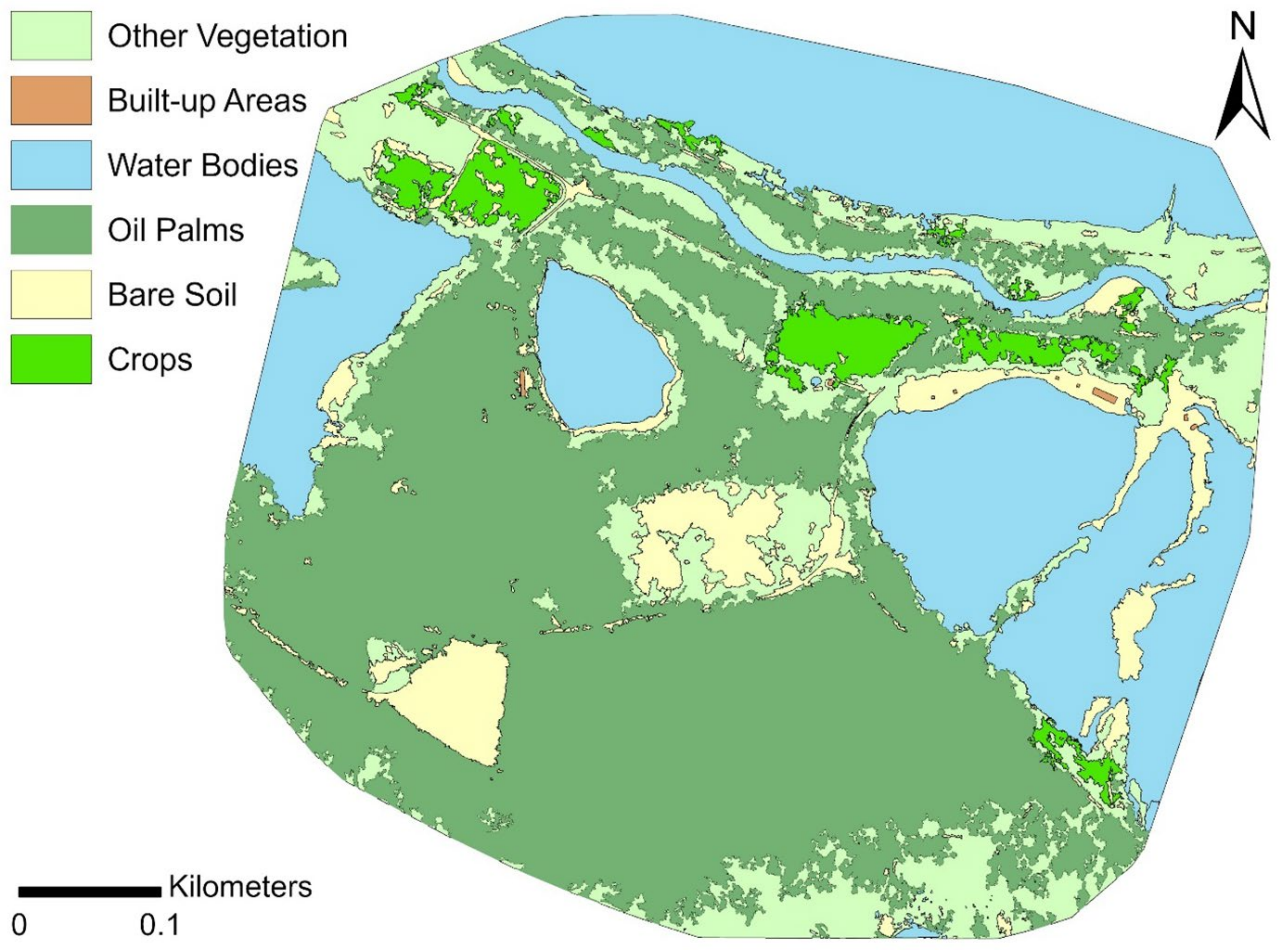
Land cover map of study area classified with OBIA method (see text for details)

A land cover map of the study area classified using the OBIA approach is shown in Fig. 7. The majority of the area was composed of oil palms and water bodies. The total area occupied by oil palm was 37 ha. Standing water bodies were mainly lakes formed in old tin mining depressions and some aquaculture ponds, whilst a single thread river channel crossed the northern part of the area. Other than oil palms and crops, vegetation included shrubs, grasses and trees, whilst bare soil included unpaved roads and unvegetated open areas where the soil was exposed. Built-up areas consisted of houses as well as buildings where domesticated livestock was housed.

Classified RGN imagery had an overall classification accuracy of 89% and a kappa value of 0.871 (Table 3), with the user and producer accuracy both similarly high (89% and 90%, respectively). The land cover classes of crops and other vegetation had the lowest user and producer accuracy, with both being 76%.

**Table 3:**
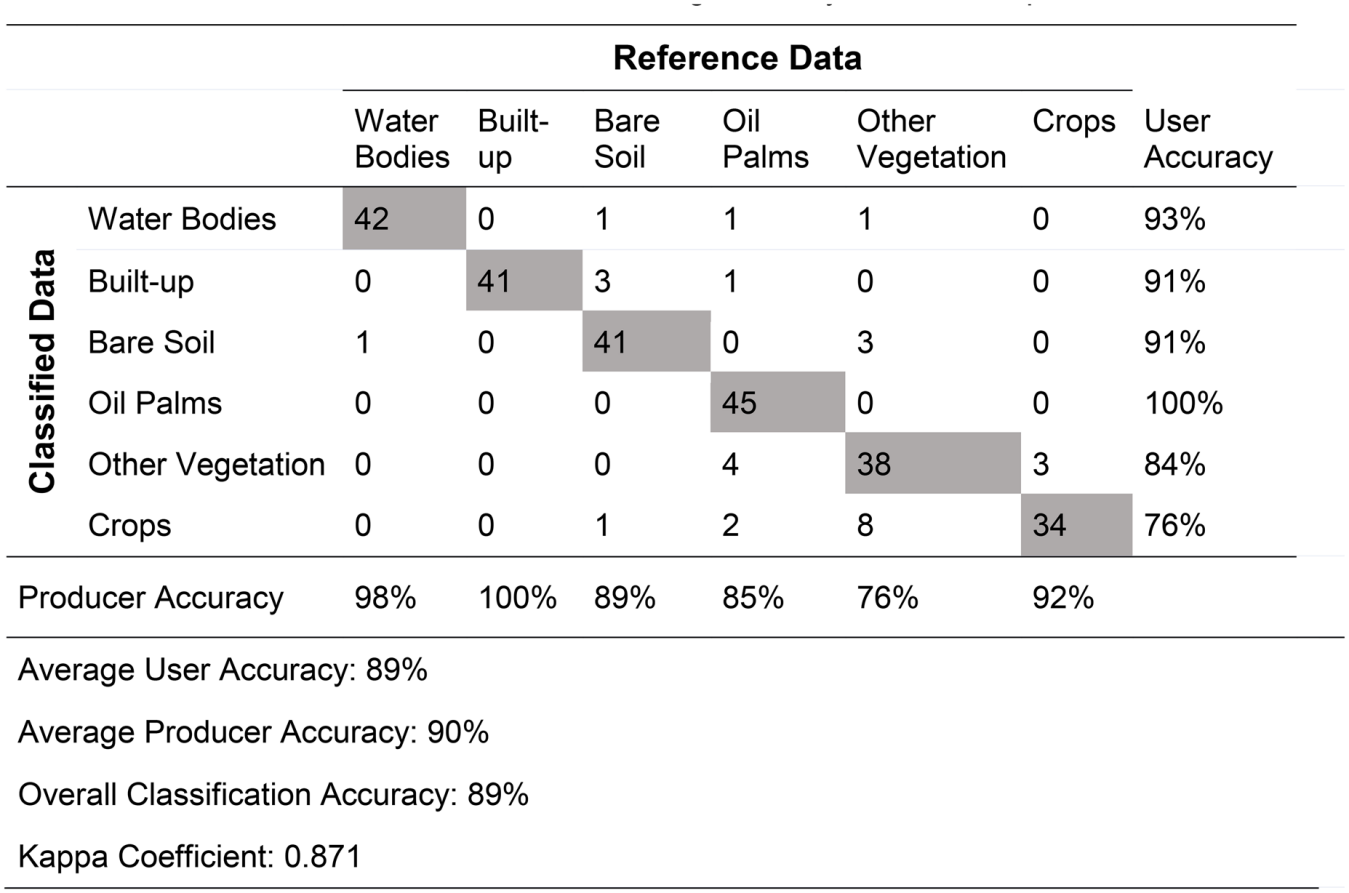
Error matrix and accuracy assessment for the OBIA classification. The original RGN orthomosaic served as reference data for validating accuracy assessment points

The bold values represent the number of validation points in the reference data which were correctly identified in the classified data

### Multi-criteria sustainability assessment

The multi-criteria analysis allowed for the production of numerous indicator maps, which were then used to generate an aggregate index of sustainability for the area. In the following sections, outputs of the sustainability assessment, including the indicator map layers and final sustainability map, are presented.

#### Indicator maps

**Fig. 8:**
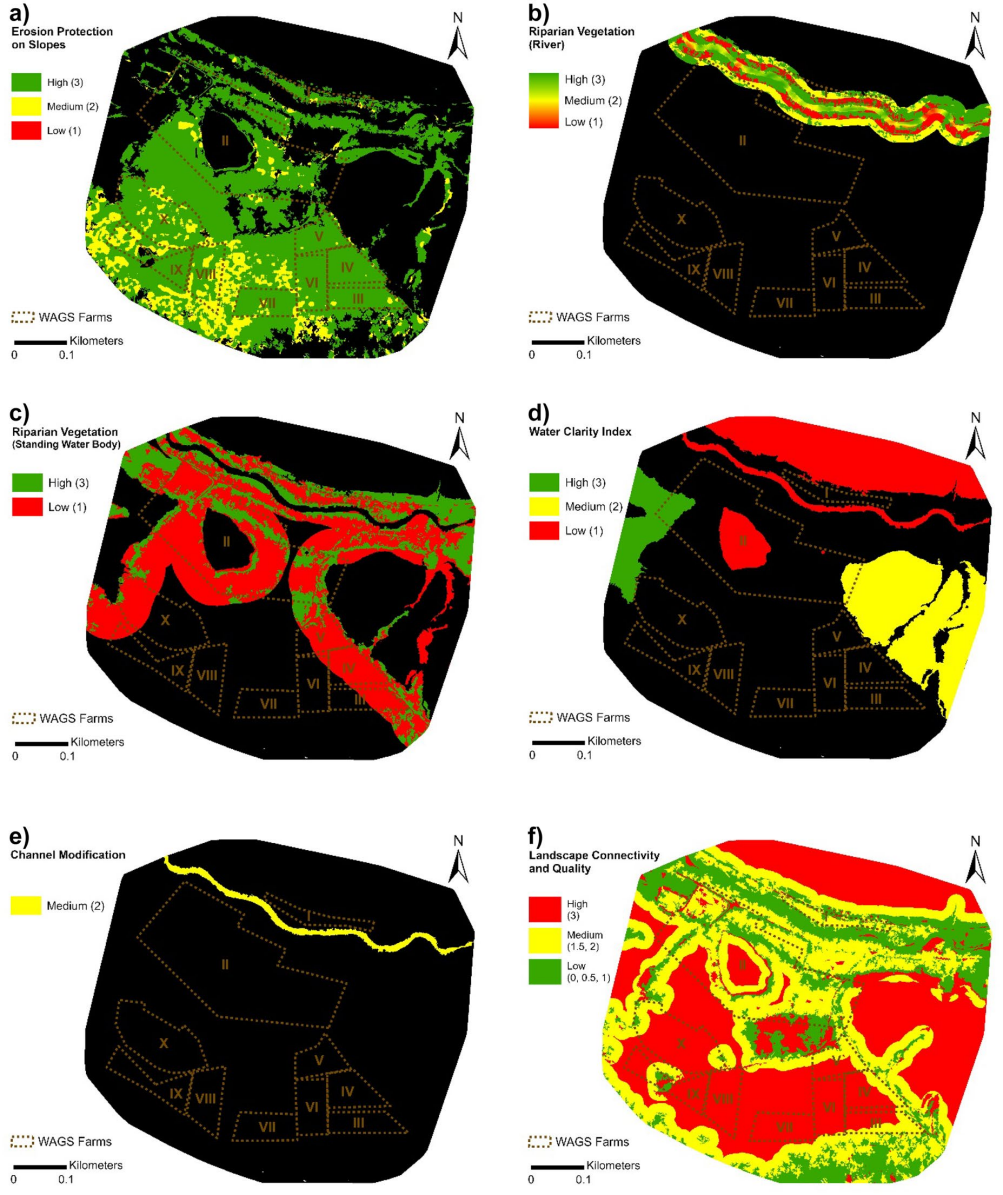
Indicator maps of environmental sustainability. Areas coloured black are those that were not included in respective indicator assessments; e.g. only water bodies were assessed for water clarity and channel modification, so all non-water areas are left black

**Fig. 9:**
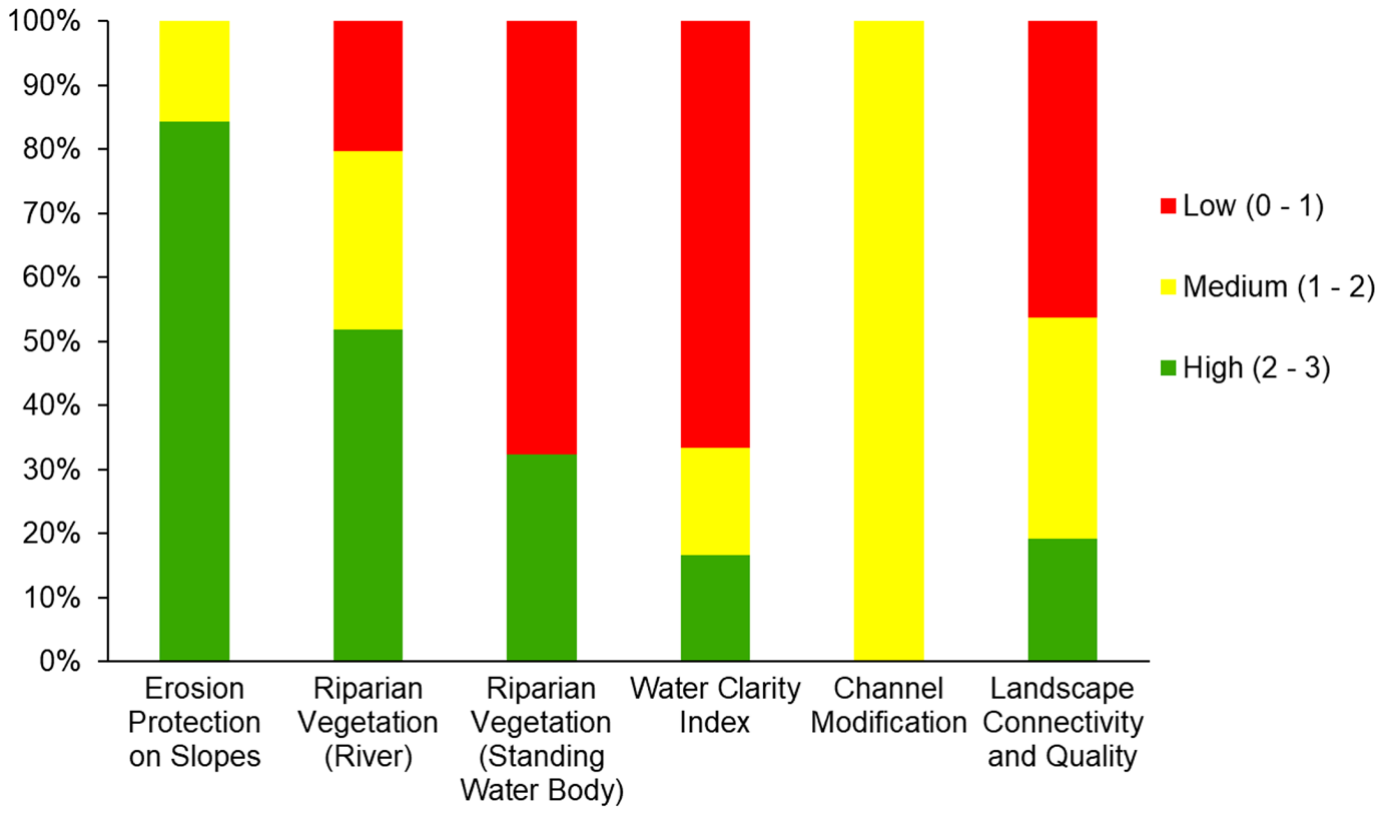
Percentage of surface of the study area falling into the three sustainability classes for each of the indicators

Areas of bare earth and oil palm were mostly on land with moderate or low slopes and therefore fell within the medium and high sustainability classes (scores 2 and 3, respectively) (Fig. 8a). Many parts of the riparian zone within the study area were classified as bare or cultivated areas and so were assigned as having a low sustainability score. The water clarity indicator highlighted that most water bodies in the area were highly turbid, with red band values skewed towards the higher of the three classes used. Sinuosity index for the main river cutting across the study area was 1.11 (see Fig. 15 of Appendix 4). Thus, the river was relatively straight and accordingly, a medium sustainability score was allocated (Fig. 8e); this reflects the likelihood that the channel has historically been straightened and realigned, with impacts on its ecological integrity. The fifth indicator showed that natural vegetation in the oil palm landscape was poorly connected (Fig. 8f), with more than 70% of the land cover and land use classified with a low or medium sustainability score for landscape connectivity (Fig. 9). The average score of individual WAGS farms for each indicator is summarised in Table 4.

**Table 4:** Average environmental sustainability score of WAGS farms. Dashed lines represent farms where indicators are not relevant since respective features are not present; e.g. farms without a river or stream channel are not scored for the riparian vegetation buffer, whilst those with no river or standing water are not scored for water clarity. The Channel Modification indicator is not included since although the river is present in the study area it does not cross any of the WAGS farms

**Indicators**	**Farm ID**									
	**I**	**II**	**III**	**IV**	**V**	**VI**	**VII**	**VIII**	**IX**	**X**
**Erosion protection on slopes**	2.95	2.93	2.95	2.99	2.99	2.93	2.91	2.63	2.80	2.82
**Riparian vegetation (river)**	1.93	2.27	-	-	-	-	-	-	-	-
**Riparian vegetation (standing water body)**	1.55	1.53	1.01	1.00	1.06	1.00	-	-	1.00	1.11
**Water clarity index**	1	1.55	-	-	-	-	-	-	-	3
**Landscape connectivity and quality**	1.86	1.62	1.16	0.91	1.21	0.59	0.56	0.58	0.55	0.88

#### Final sustainability map

**Fig. 10:**
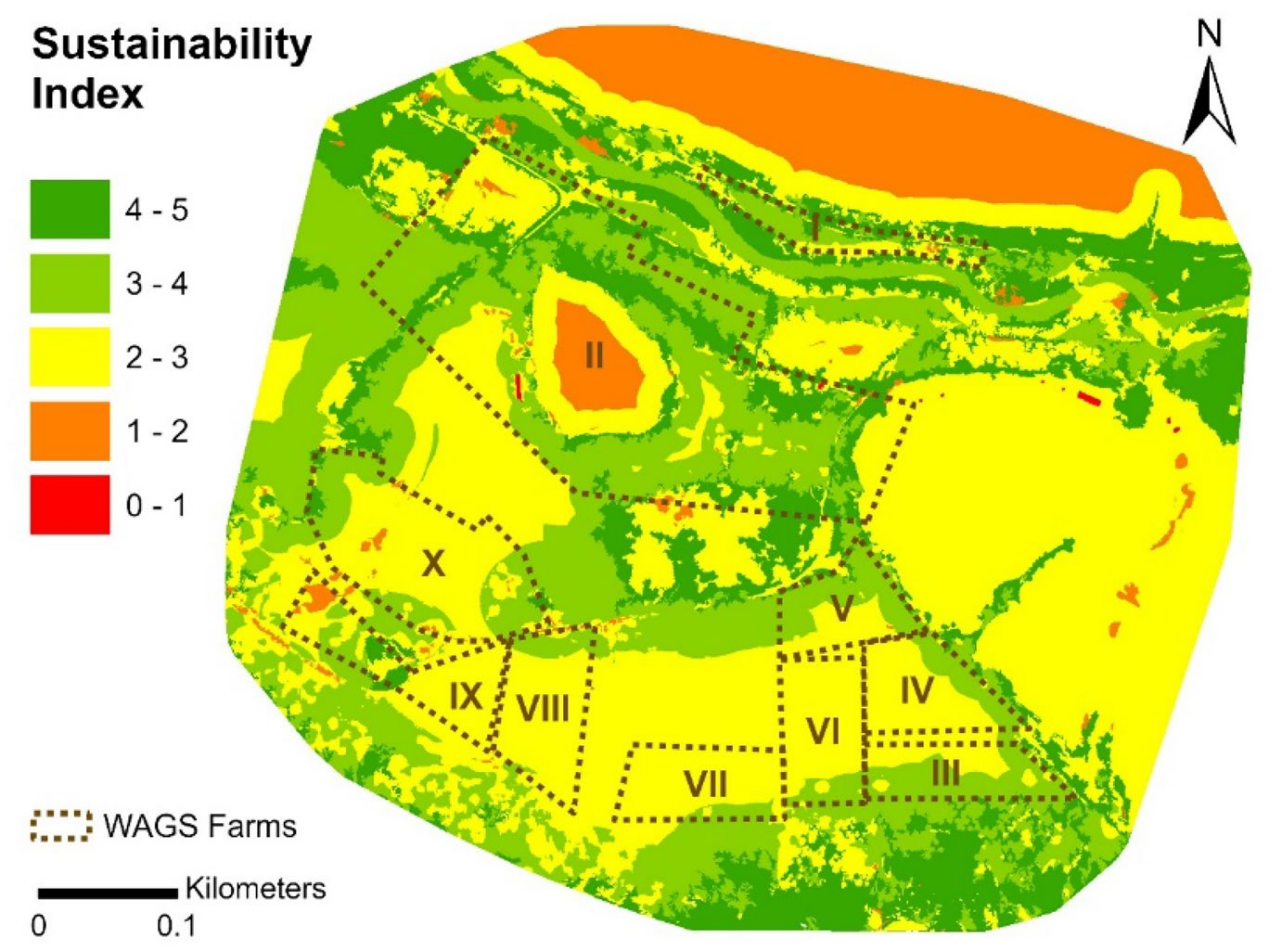
Map sustainability index scores for the study area and farms

**Fig. 11:**
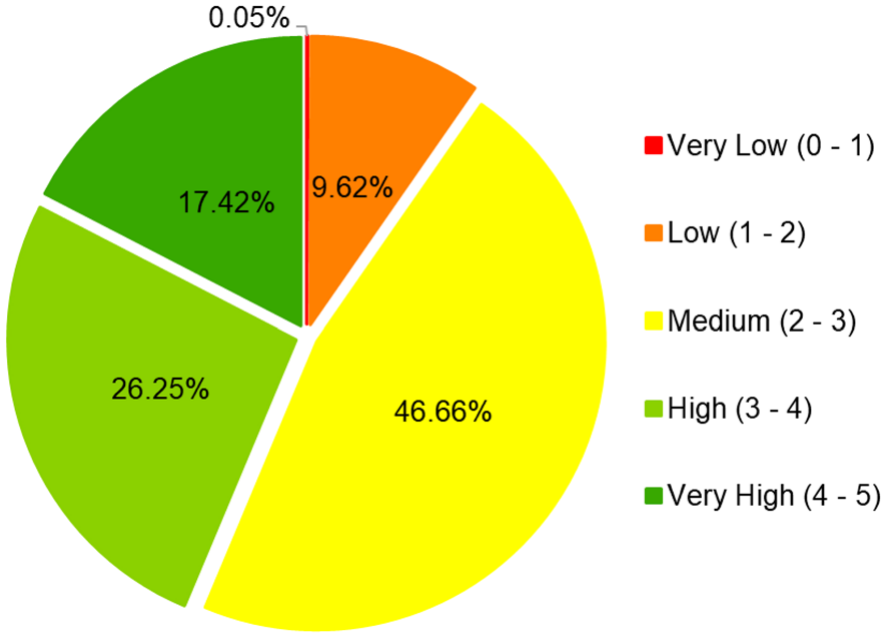
Percentage of surface of the study area falling into each sustainability class

**Fig. 12:**
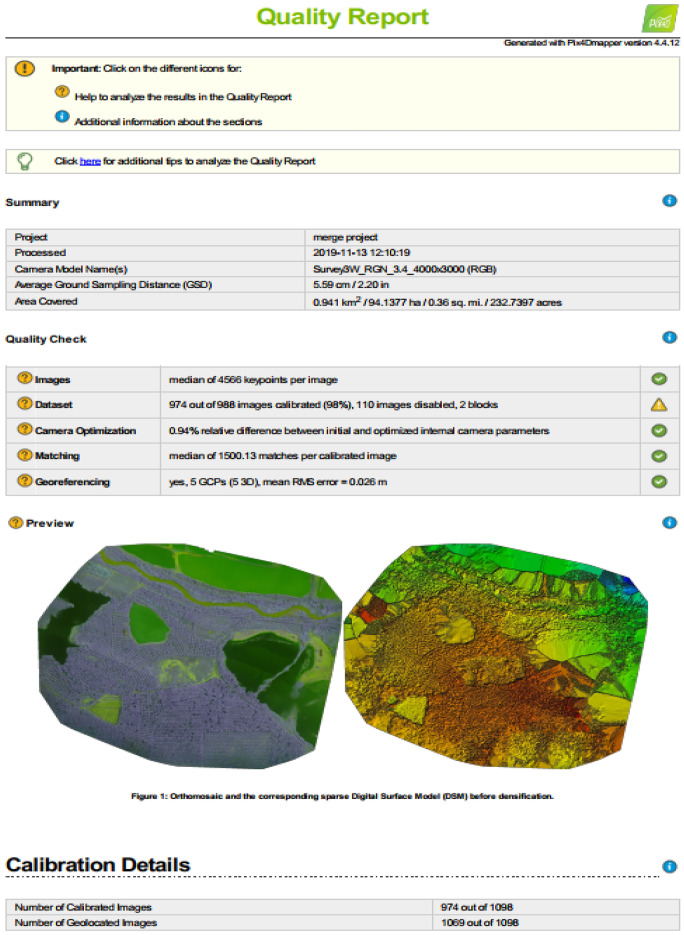
Quality report for the surface reconstruction of RGN image dataset (double-click the figure for detailed information)

**Fig. 13:**
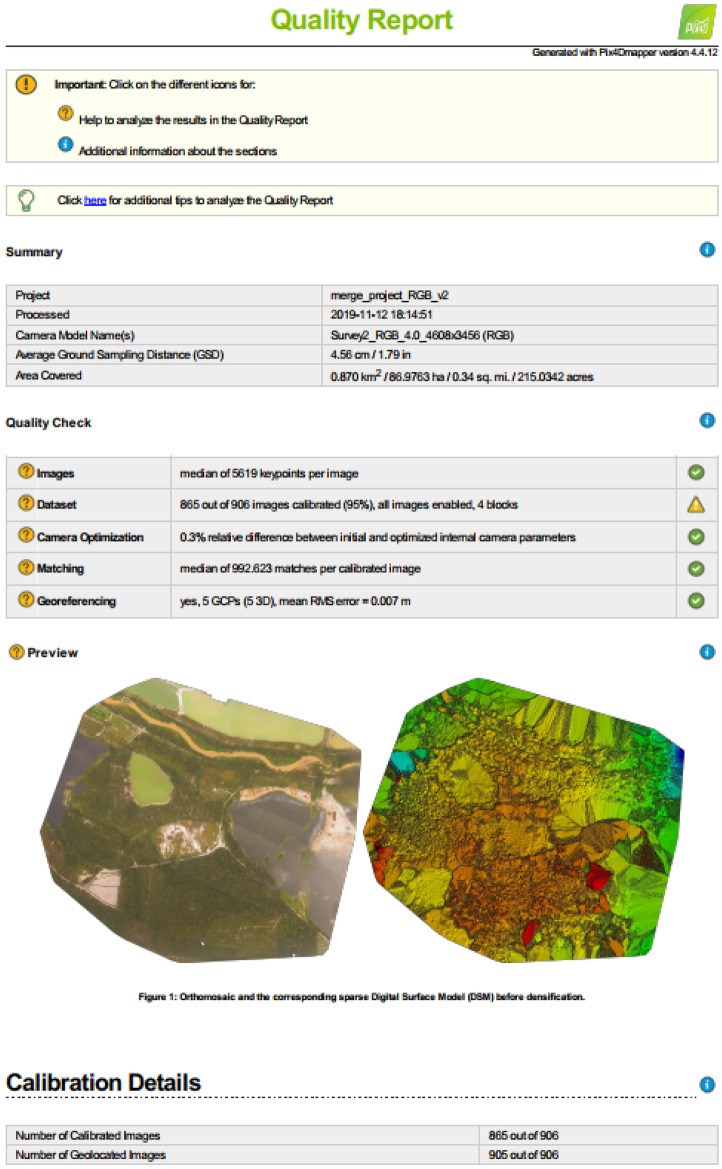
Quality report for the surface reconstruction of RGB image dataset (double-click the figure for detailed information)

**Fig. 14:**
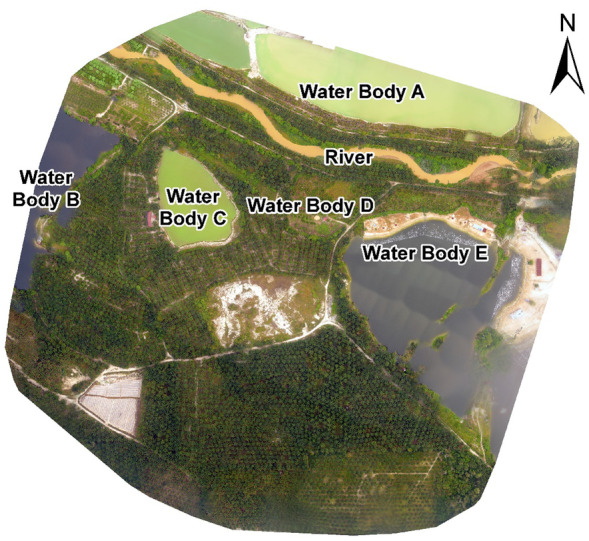
Six water bodies in the study area

**Fig. 15:**
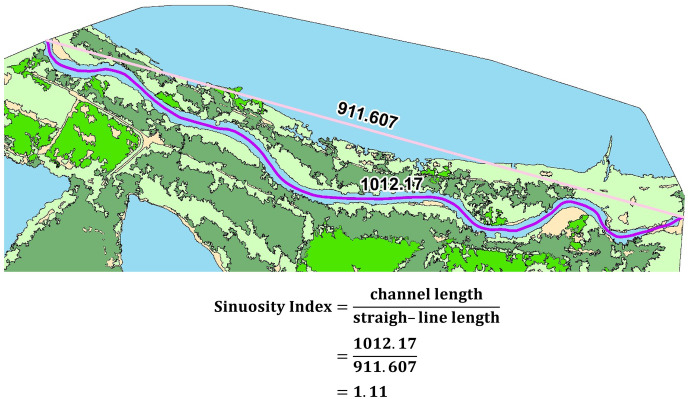
The real length and direct length of the river were first measured, and a sinuosity index was calculated through dividing the channel length (1012.17 m) by the straight-line length (911.607 m). A value of 1.11 was obtained for the sinuosity index

Values for the 5 indicators were combined to generate a final sustainability map (Fig. 10). For easy depiction and interpretation, the initial index scores for this map were reclassified to yield the 5 classes shown. The map depicts spatial variability in sustainability across the study area and within the smallholder farms. Approximately 43% of the study area had high sustainability values (Fig. 11), with 46% a medium and 9% a low score. WAGS farms mostly had moderate scores (Table 5). The median score of WAGS farms was within the medium sustainability class (between 2 and 3).

**Table 5:**
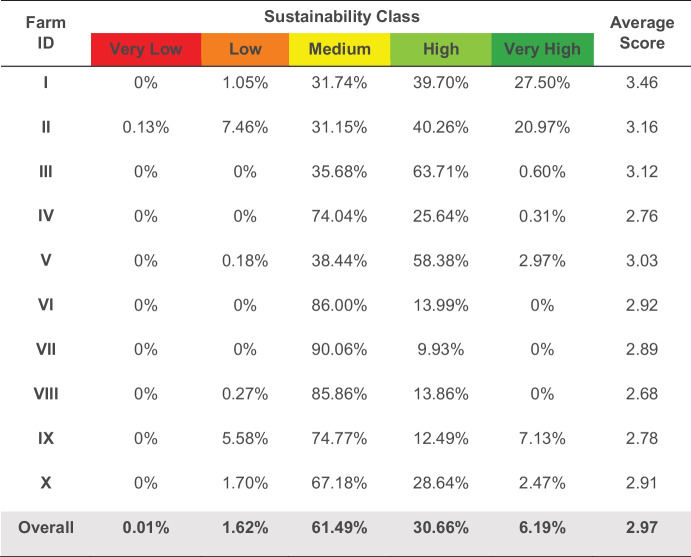
Overall sustainability index scores for the WAGS farms. Values show percentage areas of each farm falling into the various index categories (very high to very low)

## Discussion

Remote sensing technology is used widely for mapping land cover and assessing land cover change (Chong et al., 2017; Nex et al., 2022; Yao et al., 2019). UAV-derived imagery provides higher spatial resolution and accuracy for site scale applications so it represents a useful alternative to satellite-based analyses. The UAV-derived RGN and RGB imagery of our study area had pixel sizes of 0.055 m and 0.045 m and RMSE values of 0.026 m and 0.007 m respectively. The land cover mapping applied using the OBIA-based method resulted in an overall classification accuracy of 89%, with the user and producer accuracy both similarly high (89% and 90% respectively). However, the land cover classes of crops and other vegetation had lower user and producer accuracy of 76%. The UAV data were used as the basis for a multi-criteria analysis, building on existing applications of GIS environmental spatial planning and management (Aguilar-Rivera, 2019; Boggia et al., 2018; Cinelli et al., 2014; Furlan et al., 2018) to oil palm sustainability assessments at the site-scale.

Our multi-criteria analysis approach provided an array of key environmental indicators to assess the sustainability of oil palm plantations within the study area. Both the individual and combined sustainability indicators can be used in an assessment, representing a multi-dimensional approach, derived from a set of indicators, some of which are distinct and some interrelated (Milutinović et al., 2014; Mohamadzadeh et al., 2020; Singh et al., 2012). The final sustainability map suggested that 55% of the study area had low and medium sustainability scores, representing areas should be prioritised for management. The analysis revealed that areas of bare earth and oil palm were mostly found in areas with moderate or low slopes and therefore posed a low risk of erosion; these areas were therefore scored as having high sustainability index values. Conversely, many parts of the riparian zone were bare or cultivated areas and so were assigned a low sustainability score. The poor condition of the river was also reflected in its straightness, which using the sinuosity values was scored as medium. The analysis showed that natural vegetation was fairly poor connected, with more than 70% of the study area having low or medium landscape connectivity scores.

### Implications and application of the multi-criteria analysis for plantation management

The multi-criteria analysis provided information about the spatial distribution of sustainability within smallholder farms, enabling farmers to formulate sustainable management strategies. Specifically, the maps can be used to identify priority areas, such as areas with low and medium sustainability scores, where improved management is needed. Based on an interpretation of the sustainability scores, the primary recommendation to come from the work presented here is the restoration of a continuous riparian buffer strip, to be replanted with diverse range of tree species (Correll, 2005; Luke et al., 2019) and excluding oil palms. This will prevent erosion and improve water quality and, if complex vegetation structure is present, these areas will support a rich assemblage of species (Juen et al., 2016; Luke et al., 2019). The restored and existing riparian areas will need maintenance such as regular weed clearance to ensure their integrity (Barclay et al., 2017).

A second recommendation relates to bare ground. The parts of the study area with medium sustainability values included locations with bare ground; this will likely lead to soil erosion and soil loss. Ground cover management such as frond stacking and mulching therefore needs to be applied here to reduce erosion (Rahman et al., 2021; Woittiez et al., 2018). Unpaved roads should be covered with ground stones and with ground cover along the edges (Wild Asia, 2018). In the longer term, the lack of landscape heterogeneity could be addressed to support biodiversity and connectivity, such as through increasing vegetation ground cover to enhance local-scale heterogeneity and facilitate movements across the agricultural land cover matrix. The biodiversity benefits of plantations can be further improved by having a multi-strata vegetation cover that creates micro-climates and habitats (Azhar et al., 2014, 2015).

The approach presented in our study represents a novel application of multi-criteria GIS analysis to the agricultural sector, which typically focuses on assessing the suitability of the land for prospective agricultural production rather than sustainability (Feizizadeh & Blaschke, 2013; Herzberg et al., 2019; Ozsahin & Ozdes, 2022; Ustaoglu et al., 2021). Further refinements of the method particularly of the criteria weighting should be considered. The weighted linear combination method used in our study is a compensatory evaluation method (Malczewski, 2004; Masoudi et al., 2021) that allows for trade-offs between criteria, and is commonly used to support complex decision-making (Ghajari et al., 2017; Malczewski, 2004; Pérez et al., 2005). However, compensatory methods allow for better performance in one criterion to compensate for poor performance in another, which may result in unintended trade-offs (Esmail & Geneletti, 2018). For example, oil palm land managers may not want under any circumstances for riparian areas to be cleared, and high values in other criteria should not be a substitute. The weighting of different criteria based on their importance should also be considered to ensure that the most important criteria make the greatest contribution to sustainability scores (Geneletti, 2019). It is recommended that key stakeholders and experts in the oil palm sector work together to find thresholds that account for the downsides of compensatory approaches and review the relative weights for each indicator.

### Effectiveness of drone mapping and object-based classification

Underpinning the multi-criteria analysis was an accurate classified UAV-derived orthomosaic and DEM. The imagery showed good positional accuracy, with RMSE values of less than 1 pixel, which is considered to be the accuracy threshold for georeferenced and orthorectified imagery (Thomlinson et al., 1999). The good quality of our imagery was also a property of the good weather conditions (i.e. good illumination and low wind), the quality of the sensor and high-quality RTK GPS-derived GCPs (e.g. number and distribution of GCPs) (Koci et al., 2017; Seifert et al., 2019). Nevertheless, the larger water bodies in the study area were challenging for the stitching process, due to their homogeneous surface.

The OBIA classification yielded a reasonably high level of accuracy, exceeding the minimum thresholds of 85% overall and 70% per-class accuracy suggested by Thomlinson et al. (1999). However, even with the use of texture analysis and a high-quality DEM, image classification of vegetation cover across the smallholdings proved to be challenging due to the lack of spectral contrast between vegetation types, with confusion between crops and other vegetation and, as a result, a relatively low user accuracy. Such challenges associated with separating vegetation types are well known; the application of the OBIA approach with high-resolution imagery is important for addressing this challenge, as the increase in spectral variance within land cover classes associated with high-resolution pixel-based approaches makes spectral separation between land cover classes difficult (Blaschke et al., 2014; Laliberte & Rango, 2009; Marceau & Hay, 1999).

Most remote sensing studies to date have focused on surveying industrial plantations on a regional and national scale (Miettinen et al., 2017; Nomura & Mitchard, 2018; Shaharum et al., 2020; Xu et al., 2020), but large-scale mapping of oil palms is prone to missing smaller plantings (Descals et al., 2021; Rodríguez et al., 2021). The approach outlined here addresses this problem, and demonstrates the feasibility of UAV-based mapping to accurately assess smallholdings at a local level. Furthermore, few studies have investigated the potential conservation values of smallholdings which are often characterised by their heterogeneous landscapes (Azhar et al., 2011, 2013) which can only be captured by high-resolution mapping.

Mapping and monitoring of oil palm areas are essential for understanding the spatio-temporal dynamics of these landscapes (i.e. expansion and shrinkage) which, in turn, can provide useful insights into environmental impacts (Meijaard et al., 2020; Xu et al., 2020). However, financial constraints and inadequate availability of cloud-free satellite imagery have hampered land cover mapping in tropical countries (Koh & Wich, 2012; Miettinen et al., 2017; Xu et al., 2020). The present study demonstrates the utility of UAVs as a low-altitude (i.e. below cloud) remote sensing platform to provide solutions for monitoring oil palm plantations in tropical countries. Our methods provide a flexible and cost-effective remote sensing platform by using a UAV fitted with two off-the-shelf sensors. The approach is especially useful for Global South countries where labour is relatively cheaper than the purchase cost of high-resolution earth observation imagery (compared to the Global North). The greatest challenge is building technical capacity to apply such approaches, and therefore, further development of standardise workflows and guidance is required to operationalise such approaches in countries in the Global South.

Whilst UAV remote sensing is considered the future of precision agriculture (Aslan et al., 2022; Chong et al., 2017; Liu et al., 2022), there are a number of challenges which could be addressed through future research. Firstly, developing methods to systematically select the segmentation parameters and classification approach (i.e. application of texture analysis), which are usually determined through a trial-and-error approach, requiring prior knowledge and experience (Arvor et al., 2013; Feng et al., 2015), and are dependent on the image resolution and objects mapped (Bao et al., 2014; Gu et al., 2017; Ma et al., 2015). Secondly, using sensors other than the most frequently used RGB cameras (Tsouros et al., 2019; Yao et al., 2019), such as multispectral, hyperspectral and LiDAR which have been shown to be beneficial for monitoring agricultural environments (Liu et al., 2022; Nex et al., 2022; Shamshiri et al., 2019) would bring advantages. However, aside from the size and weight constraints (Colomina & Molina, 2014; Liu et al., 2022), these sensors are often not affordable for wider applications (Shamshiri et al., 2019; Tsouros et al., 2019; Yao et al., 2019). In addition, these sensors require a complex pre-processing procedure for extracting ready-to-use information (Colomina & Molina, 2014; Nex et al., 2022; Tsouros et al., 2019). Operationalising UAVs for smallholder sustainability mapping will require a solution to these challenges and balance technical capabilities, cost and accuracy.

## Conclusions

This study represents a proof of concept for the utility of UAVs for undertaking sustainability assessments of oil palm plantations. It demonstrated the utility of UAV-derived high-resolution imagery processed with the SfM, OBIA and texture analysis for classifying biophysical features and broad vegetation cover classes for smallholder plantation areas. Such an approach can be developed in conjunction with precision agriculture to support more efficient agricultural production. Future work to build on the sustainability assessment could also include other indices, including wider aspects of water quality and socio-economic indicators. This work needs to be conducted in consultation with the oil palm industry, to integrate individual indicators and/or improve on the weightings used here, so as to better support certification schemes such as the RSPO. Systematic workflows and guidance can also be developed related to optimal segmentation and classification approaches for UAV image analysis. Research and development could also be directed towards miniaturising advanced imaging sensors and making them affordable for wider agricultural needs and applications.

## Data Availability

Data will be provided on request. We can't make the data freely available due to privacy concerns of the small holders.

## Appendix 1: Quality report for assessing photogrammetric image processing

A comprehensive report provided by the Pix4D was used to assess the quality and accuracy of the surface reconstruction of UAV-derived RGN and RGB imagery (Figs. 12 and 13).

## Appendix 2: Sustainability metrics for assessing oil palm plantations

The sustainability metrics (Table 6) were mainly derived from RSPO (2018) criteria and relevant to various different land cover types.

**Table 6:** Summary of the sustainability metrics for assessing the land cover and land use of oil palm plantations

							**Land cover types**				
							**Water bodies**	**Forests**	**Oil palm areas**	**Built-up areas**	**Bare soil**
Attributes	**Habitat characteristics**	**Landscape characteristics**	Distance of plantation areas to natural landscape (e.g. forests, water bodies, riparian habitats); cumulative area of natural forest patches; isolation of plantation areas from continuous forest; distance to modern infrastructures (e.g. roads, fences, housing settlements)				**X**	**X**	**X**	**X**	**X**
		**Vegetation structure**	**Oil palm stand structure**		Polyculture with crops; mixed-age oil palm stands with multi-strata canopy; percentage of canopy cover; height of oil palm; number of dead standing oil palm; number of dead fallen oil palm				**X**		
			**Understory vegetation structure**		Percentage and height of vegetation cover; percentage of grass cover; percentage and height of non-grass cover						
			**Native crop trees**		Density and diversity of crop species						
			**Epiphytes**		Reduce percentage of epiphyte persistence						
		**Sinuosity index**	Sinuosity = (channel length/valley length)				**X**				
		**In-stream habitat heterogeneity**	Number of flow types; number of geomorphic units (e.g. riffles and pools, point bar, waterfalls) and vegetation/wood				**X**				
	**Buffer area**	**Riparian buffer width (m) = multiples of channel width**					**X**				
		Buffer width of **100 m** *** Other permanent water bodies (e.g. lake/pond) and wetlands**					**X**				
		Buffer width of **50 m** between oil palm farm and neighbouring forest area						**X**	**X**		
	**Fragile Soil**	Minimise soil erosion through practices like ground cover management, terracing and road maintenance; no extensive planting on steep terrain						**X**	**X**	**X**	**X**
		Improve soil fertility through good agricultural practices (e.g. nutrient and biomass recycling strategy, optimal use of inorganic fertilisers); proper chemical and fertiliser storage with waste management plan							**X**		
	**Peatland**	Peatland is inventoried and protected; no new planting and construction on peat after November 2018						**X**	**X**	**X**	**X**
		Implement “Best Management Practices (BMPs) for existing oil palm cultivation on peat”; maintain at least 35 cm water level below ground; phasing out oil palm cultivation at least 40 years							**X**		
	**Slope**	**No oil palm cultivation on steep slopes; protect steep slope through ground cover management**				**Low hills (0 ~ 10°)**			**X**		**X**
						**Moderate hills (10 ~ 25°)**					
						**Steep hills (> 25°)**					
	**Primary forest**	No clearing of primary forest after November 2005						**X**			
	**High carbon stock (HCS) forest**	**Identify HCS forest; protect, enhance and no land clearing of HCS forest**		**Dense forest**		Estimated carbon > **75t/ha**, dominated by trees with **DBH > 30 cm**, more than **30 trees/ha** with DBH > 30 cm, canopy closure > **50%**		**X**			
				**Young regenerating forest**		Estimated carbon **35–75t/ha**, dominated by trees with **DBH 10 ~ 30 cm**, **15 ~ 30 trees/ha** with DBH > 30 cm, canopy closure **30 ~ 40%**					
	**High conservation values (HCVs) area**	Identify HCVs area; integrate management plan to protect or enhance HCVs					**X**	**X**			

Biophysical features bold represent key figures used to measure sustainability

## Appendix 3: Mean red band value for assessing water clarity

Six water bodies were identified in the study area (Fig. 14), and the red band value from RGB imagery was used as a representative parameter for assessing water clarity (Table 7).

**Table 7:** Mean red band value of water bodies from RGB imagery

	**Mean red band value ± SD**	**Value**
**River**	179.5 ± 53.1	Maximum
**Water body A**	160.2 ± 77.1	
**Water body B**	67.1 ± 55.0	Minimum
**Water body C**	170.6 ± 9.5	
**Water body D**	177.4 ± 28.9	
**Water body E**	128.3 ± 52.0	

## Appendix 4: Calculation of sinuosity index

A sinuosity index was calculated to assess the level of modification of riverine habitat. The sinuosity index was calculated through dividing the observed channel length between start and endpoints by the straight-line length between them. For the example below (Fig. 15), a sinuosity index with a value of 1.11 was obtained.
